# Hospital-associated methicillin-resistant *Staphylococcus aureus*: A cross-sectional analysis of risk factors in South African tertiary public hospitals

**DOI:** 10.1371/journal.pone.0188216

**Published:** 2017-11-16

**Authors:** Liliwe L. Shuping, Lazarus Kuonza, Alfred Musekiwa, Samantha Iyaloo, Olga Perovic

**Affiliations:** 1 School of Health Systems and Public Health, University of Pretoria, Pretoria, South Africa; 2 South African Field Epidemiology Training Programme, National Institute for Communicable Diseases, National Health Laboratory Service, Johannesburg, South Africa; 3 Centre for Healthcare-Associated Infections, Antimicrobial Resistance and Mycoses, National Institute for Communicable Diseases, National Health Laboratory Service, Johannesburg, South Africa; 4 Centers for Disease Control and Prevention, Pretoria, South Africa; 5 National Institute for Occupational Health, National Health Laboratory Service, Johannesburg, South Africa; 6 Department of Clinical Microbiology and Infectious Diseases, University of Witwatersrand, Johannesburg, South Africa; Pusan National University, REPUBLIC OF KOREA

## Abstract

**Introduction:**

Hospital-associated methicillin-resistant *S*. *aureus* (HA-MRSA) remains a significant cause of morbidity and mortality worldwide. We conducted a study to determine risk factors for HA-MRSA in order to inform control strategies in South Africa.

**Methods:**

We used surveillance data collected from five tertiary hospitals in Gauteng and Western Cape provinces during 2014 for analysis. A case of HA-MRSA was defined as isolation of MRSA from a blood culture 48 hours after admission and/or if the patient was hospitalised in the six months prior to the current culture. Multivariable logistic regression modelling was used to determine risk factors for HA-MRSA.

**Results:**

Of the 9971 patients with positive blood cultures, 7.7% (772) had *S*. *aureus* bacteraemia (SAB). The overall prevalence of MRSA among those with SAB was 30.9% (231/747; 95% confidence interval [CI] 27.6%– 34.3%). HA-MRSA infections accounted for 28.3% of patients with SAB (207/731; 95% CI 25.1%– 31.7%). Burns (adjusted odds ratio [aOR] 12.7; 95% CI 4.7–34.4), age ≤1 month (aOR 8.7; 95% CI 3.0–24.6), residency at a long-term care facility (aOR 5.2; 95% CI, 1.5–17.4), antibiotic use within two months of the current SAB episode (aOR 5.1; 95% CI 2.8–9.1), hospital stay of 13 days or more (aOR 2.8; 95% CI 1.3–5.6) and mechanical ventilation (aOR 2.2; 95% CI 1.07–4.6), were independent risk factors for HA-MRSA infection.

**Conclusion:**

The prevalence of MRSA remains high in South African tertiary public hospitals. Several identified risk factors of HA-MRSA infections should be considered when instituting infection and prevention strategies in public-sector hospitals, including intensifying the implementation of antimicrobial stewardship programmes. There is an urgent need to strengthen infection prevention and control in burn wards, neonatal wards, and intensive care units which house mechanically ventilated patients.

## Introduction

Nosocomial infections are a significant cause of morbidity, mortality, and excess healthcare costs globally [1]. *Staphylococcus aureus* is the most common human pathogen causing both community- and hospital-associated infections, including pneumonia and bacteraemia [2]. Hospital-associated methicillin-resistant *S*. *aureus* (HA-MRSA) accounts for a high proportion of hospitalised patients infected with *S*. *aureus* [3]. In most African countries, including South Africa, HA-MRSA constitutes 20–50% of *S*. *aureus* infections [3].

Since its emergence in the 1960s and recognition as a nosocomial pathogen, MRSA remains a significant cause of illness among the population today [3]. Compared to infections with methicillin-sensitive *S*. *aureus* (MSSA), methicillin-resistant *S*. *aureus* (MRSA) infections are associated with higher morbidity and mortality [4]. A meta-analysis found that the risk of death due to MRSA bacteraemia was two times higher compared to MSSA bacteraemia [5]. MRSA infections also result in longer hospital stays compared to MSSA infections [6]. This increases utilization of hospital resources such as medication and additional staff, resulting in 1.3 to 3 times higher cost of treatment [7,8].

In order to control HA-MRSA infections, it is important to understand the burden of disease and risk factors associated with infection. Identification of risk factors for HA-MRSA is essential as it guides infection control and prevention policies, guidelines and related activities. In South Africa, there are limited reports on HA-MRSA and its associated risk factors. We aimed to describe the epidemiology of HA-MRSA and identify risk factors in comparison to HA-MSSA in five public sector hospitals in South Africa.

## Methods

### Study design and setting

We conducted a cross-sectional analysis on demographic, laboratory and clinical data collected through GERMS-SA, an active laboratory-based surveillance system for selected pathogens of public health importance. All hospitals taking part in the surveillance program and included in our study were tertiary academic facilities with diagnostic stewardship programs that includes clinical microbiology consultancy on specimen submission practices as one of the components. All patients presenting with clinical signs and symptoms such as temperature >38°C, increased white cell count, elevated C-reactive protein, erythrocyte sedimentation rate, urea and electrolytes, and lung abnormalities on a chest X-ray, qualify for a blood culture. Five tertiary public-sector hospitals, including Helen Joseph Hospital (HJH), Steve Biko Academic/Tshwane District Hospital (SBH), Charlotte Maxeke Johannesburg Academic Hospital (CMJAH), Groote Schuur Hospital (GSH) and Tygerberg Hospital (TBH) participated in the GERMS-SA surveillance system during 2014. HJH (900 beds), SBH (832 beds) and CMJAH (900 beds) are in Gauteng Province, and GSH (975 beds) and TBH (1,384 beds) are in the Western Cape Province. These hospitals are affiliated with universities and serve mainly urban populations.

### Data collection and laboratory testing

Patient level and laboratory data were obtained from the GERMS-SA electronic database. GERMS-SA surveillance officers interviewed consenting patients who had *S*. *aureus* bacteraemia (SAB) and captured data using standard case report forms. For patients who refused to give consent, only medical and laboratory records were used to obtain data. Isolates from these patients were submitted to the Antimicrobial Resistance Reference Laboratory at the National Institute for Communicable Diseases for confirmatory identification and antimicrobial susceptibility testing. Isolate identification was done using the Matrix-Assisted Laser Desorption/Ionization Time-of-Flight Mass Spectrometry (MALDI-TOF MS) Flex analysis system, version 3.4 (Bruker Daltonics, Germany). Antimicrobial susceptibility testing was done using the automated Microscan Walkaway system (Beckman Coulter Inc.) and minimum inhibitory concentrations were interpreted using Clinical and Laboratory Standard Institute guidelines [9]. Data were entered or imported into the GERMS-SA electronic database. A proportion of SAB cases had no isolates submitted to the surveillance laboratory as they were identified during quarterly audits done by GERMS-SA. Audits were performed to detect missed cases using data from the National Health Laboratory Service Corporate Data Warehouse database, which houses routine laboratory test results for public-sector hospitals in South Africa. For such cases, antimicrobial susceptibility results used in the current study were those obtained from the hospitals’ laboratory reports. We obtained data on total blood cultures performed at sentinel sites from the National Health Laboratory Service Corporate Data Warehouse.

### Study population and case definition

A case of SAB was defined as a *S*. *aureus* positive blood culture isolated between 1^st^ January 2014 and 31^st^ December 2014 from patients admitted to any of the sentinel sites. Where a patient had additional isolates within 21 days of the first positive culture, only the first episode was included. MRSA was defined as non-susceptibility to oxacillin. Hospital-associated infection was defined as collection of a positive blood culture more than 48 hours after admission, or if a patient was hospitalised within a six-month period preceding the positive culture. SAB cases occurring within 48 hours of admission with no prior hospitalisation in the preceding six months were considered community-associated infections, and were not included in our analysis for risk factors.

### Data analysis

To calculate positivity rates for *S*. *aureus*, the number of blood cultures positive for *S*. *aureus* was divided by the total number of blood cultures performed at hospital laboratories (one culture per patient per day). To determine the proportion of patients with SAB among patients with bloodstream infections (patients with a positive blood culture for any bacterial organisms, de-duplicated in the same manner as SAB cases), the number of SAB cases was divided by the total number of positive cultures. The corresponding 95% confidence intervals (CI) were also calculated for positivity rates and for the proportions. Descriptive statistics were used to describe characteristics of HA-MRSA and HA-MSSA cases. Associations between categorical variables were tested using Chi-square tests. Differences in non-normally distributed continuous variables between two groups were tested using the non-parametric Wilcoxon rank-sum test. Logistic regression was used to determine risk factors for HA-MRSA infection. The potential risk factors analysed included demographic characteristics, clinical characteristics such as presence of mechanical ventilation and central venous catheters, and underlying conditions such as heart disease, diabetes mellitus, smoking and malignancy. All variables with a p-value of ≤ 0.2 in the univariate analyses were entered into the multivariable model using the manual forward stepwise selection. Multi-collinearity tests were performed before entering variables in the multivariable model to ensure that only non-collinear variables were included. Variables with a p-value of ≤ 0.05 were kept in the model. The final model was tested using the Hosmer-Lemeshow Chi-square goodness-of-fit test. All statistical analyses were performed using STATA statistical software (Version 13; StataCorp LP Texas USA) and a p-value of ≤ 0.05 was considered statistically significant.

### Ethics

Ethical approval for this study was obtained from the Faculty of Health Sciences Research Ethics Committee of the University of Pretoria (458/2015). The protocol was also approved by the U.S Centers for Disease Control and Prevention (CDC)’s Human Subjects Research Office. Ethics approval for the GERMS-SA enhanced *S*. *aureus* surveillance was granted by the Human Research Ethics Committee (Medical) of the University of Witwatersrand (No: M10464). Permission to use data from the National Health Laboratory Service Corporate Data Warehouse was obtained from the National Health Laboratory Service Research and Academic Committee. Extracted data were anonymised and stored on password protected computers.

## Results

### Rates of *S*. *aureus*

**Fig 1:**
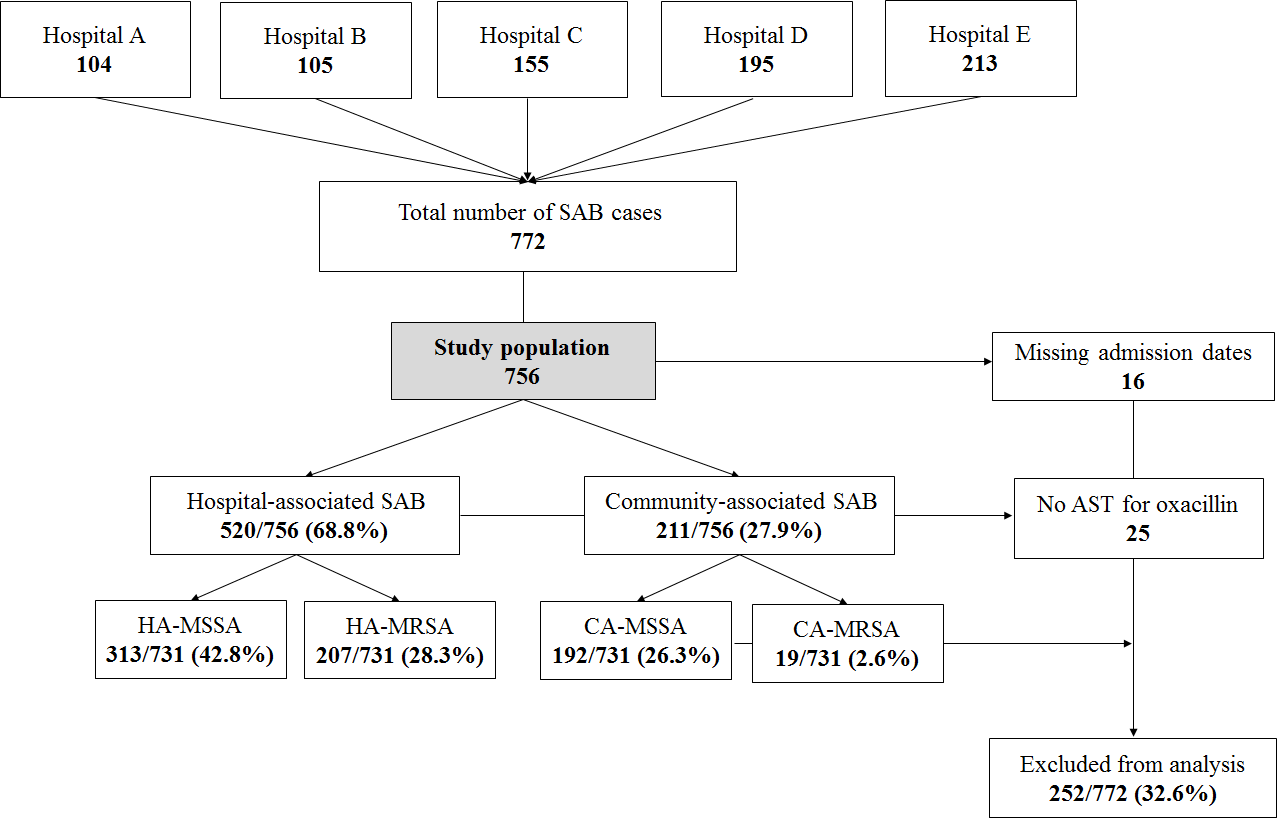
The number of SAB cases at five hospitals in Gauteng and Western Cape during 2014. AST, antimicrobial susceptibility testing results; SAB, *S*. *aureus* bacteraemia; CA, community-associated; HA, hospital-associated; MSSA, methicillin sensitive *S*. *aureus*; MRSA, methicillin-resistant *S*. *aureus*.

There were 772 SAB cases diagnosed at the five sentinel sites during 2014 (Fig 1). Of these patients, 68.8% (520/756) had hospital-associated infections and 27.9% (211/756) had community-associated infections. Among SAB cases who had oxacillin susceptibility results, the overall prevalence of MRSA was 30.9% (231/747; 95% CI 27.6%– 34.3%). HA-MRSA infections accounted for 28.3% (207/731; 95% CI, 25.1%– 31.7%) of *S*. *aureus* bloodstream infections.

*S*. *aureus* was isolated from 1.4% (876/62069; 95% CI 1.3% - 1.5%) of all blood cultures performed at sentinel sites, with ranges of 1.1%– 1.5% between hospitals (Table 1). Among patients with bloodstream infections, the proportion of SAB was 7.7% (772/9971; 95% CI 7.2%– 8.3%).

**Table 1:** Rates of SAB at five hospitals in Gauteng and Western Cape during 2014.

	Proportion of *S*. *aureus* among all blood cultures		Proportion of *S*. *aureus* cases among bloodstream infections	
Hospital	Total number of blood cultures	*S*. *aureus* positive cultures (%)	Total number of positive cultures	SAB cases (%)
**A**	9190	104 (1.1%)	1436	104 (7.2%)
**B**	9696	143 (1.5%)	2267	105 (4.6%)
**C**	13246	191 (1.4%)	1704	155 (9.1%)
**D**	13456	241 (1.8%)	1801	195 (10.8%)
**E**	16481	233 (1.4%)	2763	213 (7.7%)
**Total**	62069	876 (1.4%)	9971	772 (7.7%)

SAB, *S*. *aureus* bacteraemia.

### Characteristics of patients with HA-MRSA and HA-MSSA infections

There were 313 HA-MSSA cases and 207 HA-MRSA cases. Neonatal cases (**≤**1 month old) accounted for the highest proportion (56/207 [27.1%]; p <0.001) of HA-MRSA infections across all hospitals, while none of the patients aged 6–14 years were infected with HA-MRSA (Table 2). Antibiotic use in the previous two months before the current SAB episode was strongly associated with HA-MRSA compared to HA-MSSA infection (62.1% vs 22.5%; p <0.001). Compared to HA-MSSA cases, a higher proportion of HA-MRSA cases were mechanically ventilated (30.7% vs 14.2%; p <0.001) and had central venous catheters in place (45.5% vs 38.7%; p = 0.132) at the time of specimen collection, although the latter result was not statistically significant. A significantly higher proportion of HA-MRSA cases were disorientated (15.5% vs 8.5%; p = 0.002), stuporous (26.2% vs 12.3%; p = 0.002) or sedated (4.8% vs 3.3%; p = 0.002) compared to HA-MSSA cases. The total length of hospital stay was also significantly longer among those with HA-MRSA compared to those with HA-MSSA infections (median days: 39 vs 21; p <0.001). Lastly, a higher proportion of patients with HA-MRSA died compared to patients with HA-MSSA (38.9% vs 26.6%; p = 0.015). The proportion of cases with HIV and tuberculosis infections, those previously infected/colonised with MRSA, those currently receiving antibiotic treatment, and those with pre-existing conditions was similar between HA-MSSA and HA-MRSA infections.

**Table 2:** Characteristics of patients with HA-MRSA and HA-MSSA infections at five hospitals in Gauteng and Western Cape during 2014.

Characteristics	MSSA N = 313	MRSA N = 207	
	n (%)	n (%)	p value
Age			
≤ 1 month	12 (3.8)	56 (27.1)	**<0.001**
> 1 month—5 years	30 (9.6)	27 (13.0)	
6–14 years	15 (4.8)	0 (0)	
5–24 years	25 (8.0)	9 (4.3)	
25–34 years	54 (17.3)	14 (6.8)	
35–44 years	36 (11.5)	13 (6.3)	
45–54 years	30 (9.6)	20 (9.7)	
55–64 years	22 (7.0)	13 (6.3)	
≥ 65 years	35 (11.2)	15 (7.3)	
Unknown	54 (17.2)	40 (19.3)	
Sex			
Female	89 (28.4)	63 (30.4)	0.651
Male	170 (54.3)	104 (50.2)	
Unknown	54 (17.3)	40 (19.3)	
Hospital			
A	33 (10.5)	7 (3.4)	**<0.001**
B	50 (16.0)	21 (10.1)	
C	80 (25.6)	25 (12.1)	
D	82 (26.2)	66 (31.9)	
E	68 (21.7)	88 (42.5)	
Source of bacteraemia			
Bacteraemia without focus	207 (66.1)	145 (70.0)	**0.009**
Lower respiratory tract infection	23 (7.3)	23 (11.1)	
Cerebral spinal fluid	4 (1.3)	2 (1.0)	
Skin/soft tissue infection	49 (15.7)	32 (15.5)	
Other	30 (9.6)	5 (2.4)	
Mental status^a^			
Alert	161/212 (75.9)	45/84 (53.6)	**0.002**
Disorientated	18/212 (8.5)	13/84 (15.5)	
Stuporous	26/212 (12.3)	22/84 (26.2)	
Sedated	7/212 (3.3)	4/84 (4.8)	
Pre-disposing factors			
HIV positive	55/229 (24.0)	24/116 (20.7)	0.487
Tuberculosis	13/307 (4.2)	5/203 (2.5)	0.286
Residence at a LTCF^b^	7/302 (2.3)	15/201 (7.5)	**0.006**
Referred from a LTCF/hospital	68/306 (22.2)	60/166 (29.3)	0.060
Previous MRSA infection/colonisation^b^	11/303 (3.6)	8/197 (4.1)	0.806
Previous dialysis^b^			
Haemodialysis	28/300 (9.3)	4/203 (1.9)	**0.001**
Peritoneal	11/300 (3.7)	3/203 (1.5)	
Current dialysis			
Haemodialysis	38/312 (12.2)	4/207 (1.9)	**<0.001**
Peritoneal	13/312 (4.2)	2/207 (0.9)	
Previous surgery^b^	77/304 (25.3)	58/202 (28.7)	0.387
Current surgery	69/312 (22.0)	46/207 (22.2)	0.962
Central venous catheters^c^	117/302 (38.7)	91/200 (45.5)	0.132
Mechanical ventilation^a^	43/303 (14.2)	61/199 (30.7)	**<0.001**
Pre-existing conditions^d^	267/312 (85.6)	187/207 (87.9)	0.444
Antibiotic use			
24 hrs prior to positive culture	52/304 (17.1)	72/201 (35.8)	**<0.001**
Previous 2 months	64/285 (22.5)	123/198 (62.1)	**<0.001**
Current treatment	275/303 (90.5)	192/203 (94.6)	0.092
Length hospital stay (days)			
Before positive culture (median; IQR)	5 (0–11)	12 (9–45)	**<0.001**
After positive culture (median; IQR)	11 (5–27)	19 (7–23)	**<0.001**
Entire stay (median; IQR)	21 (10–38)	39 (19–66)	**<0.001**
Outcome			
Recovered/Discharged	221/308 (71.8)	119/198 (60.1)	**0.015**
Died	82/308 (26.6)	77/198 (38.9)	
Refused treatment	5/308 (1.6)	2/198 (1.0)	

MSSA, methicillin sensitive *S*. *aureus*; MRSA, methicillin-resistant *S*. *aureus*; LTCF, long-term care facility; IQR, interquartile range.

^a^ On the day of positive culture

^b^ Within a year of the current culture

^c^ Within 2 days of the current culture

^d^ Pre-existing conditions included chronic obstructive pulmonary diseases, chronic renal disease, cerebrovascular disease (cerebro-vascular accident, cerebral palsy, neuromuscular), heart disease, head injuries (head surgery, ventricular shunts, cochlear implants, cerebrospinal fluid leaks), connective tissue diseases, liver disease, aplastic anaemia, primary immunodeficiency conditions, immunosuppressive treatment, diabetes mellitus, malignancy, organ transplant, surgery, prematurity, protein energy malnutrition, burns, alcohol dependency, smoking, pancreatitis, dementia, obesity and decubitus/pressure ulcers.

### Risk factors analysis

In the univariate analysis, age ≤ 1 month (odds ratio [OR] 10.9; 95% CI 4.5–25.9), admission at hospital D (OR 3.8; 95% CI 1.5–9.1) and hospital E (OR 6.1; 95% CI 2.5–14.6), hospital stay of 5–12 days (OR 4.5; 95% CI 2.7–7.4), hospitals stay of ≥13 days (OR 7.2; 95% CI 4.3–12.0), burns (OR 4.7; 95% CI 2.3–9.4), mechanical ventilation (OR 2.7; 95% CI 1.7–4.1), abdominal surgery (OR 1.9; 95% CI 1.04–3.4), residence in long-term care facilities (LTCFs) (OR 3.4; 95% CI 1.3–8.3), and antibiotic use in the last two months (OR 5.7; 95% CI 3.7–8.4) were significantly associated with HA-MRSA (Table 3). Previous haemodialysis (OR 0.2; 95% CI 0.06–0.5), current haemodialysis (OR 0.1; 95% CI 0.04–0.3), current peritoneal dialysis (OR 0.2; 95% CI 04–0.8), renal disease (OR 0.2; 95% CI 0.07–0.3), having malignancies (OR 0.5; 95% CI 0.2–0.9), head injuries (OR 0.3; 95% CI 0.1–0.8), and being on immunosuppressive treatment (OR 0.3; 95% CI 0.1–0.7) reduced the odds of HA-MRSA infection. However, most of these associations were not significant in the multivariable model.

**Table 3:** Univariate analysis of risk factors for HA-MRSA infection in comparison to HA-MSSA infection among hospitalised patients in Gauteng and Western Cape during 2014.

Characteristic	OR	95% CI	p value
Age			
≤ 1 month	10.9	4.5–25.9	**<0.001**
> 1 month—5 years^a^	2.1	0.9–4.6	0.068
15–24 years	0.8	0.3–2.2	0.725
25–34 years	0.6	0.2–1.4	0.261
35–44 years	0.8	0.3–2.0	0.702
45–54 years	1.6	0.6–3.5	0.296
55–64 years	1.4	0.5–3.4	0.491
≥ 65 years	Ref		
Sex			
Male	0.9	0.5–1.2	0.480
Hospital			
A	Ref		
B	2.0	0.7–5.1	0.164
C	1.5	0.5–3.7	0.400
D	3.8	1.5–9.1	**0.003**
E	6.1	2.5–14.6	**<0.001**
LOS before positive culture (days)			
0–4	Ref		
5–12	4.5	2.7–7.4	**<0.001**
≥ 13	7.2	4.3–12.0	**<0.001**
Renal disease	0.2	0.07–0.3	**<0.001**
Burns	4.7	2.3–9.4	**<0.001**
HIV	0.8	0.4–1.4	0.487
Antiretroviral therapy	0.6	0.2–1.6	0.325
Malignancy	0.5	0.2–0.9	**0.028**
Head Injuries	0.3	0.1–0.8	**0.021**
Diabetes	0.7	0.3–1.1	0.153
Mechanical ventilation^b^	2.7	1.7–4.1	**<0.001**
Central venous catheter^c^	1.3	0.9–1.8	0.125
Immunosuppressive treatment	0.3	0.1–0.7	**0.002**
Mixed infections	1.7	0.8–3.4	0.126
Abdominal surgery	1.9	1.04–3.4	**0.035**
Non-abdominal surgery	0.7	0.3–1.1	0.144
Resident in LTCF^d^	3.4	1.3–8.4	**0.009**
Exposed to a crowded place	0.5	0.2–0.9	**0.039**
Previous antibiotic use^e^	5.7	3.7–8.4	**<0.001**
Current antibiotic treatment	1.8	0.8–3.7	0.096
Previous dialysis^d^			
Haemodialysis	0.2	0.06–0.5	**0.002**
Peritoneal dialysis	0.4	0.1–1.3	0.125
Current dialysis			
Haemodialysis	0.1	0.04–0.3	**<0.001**
Peritoneal dialysis	0.2	0.4–0.8	**0.036**
Previous infections^d^	0.5	0.1–1.5	0.246
Previous MRSA infection/colonisation^d^	1.1	0.4–2.8	0.806

OR, odds ratio; CI, confidence interval; LOS, length of hospital stay; LTCF, long-term care facility; MRSA, methicillin-resistant *S*. *aureus*.

^a^ The 6–14 years age group was omitted in the model due to zero outcome observations;

^b^ On the day of positive culture;

^c^ Within two days of the current culture;

^d^ Within a year of the current culture;

^e^ Two months prior the current culture.

In the multivariable analysis, burns (aOR 12.7; 95% CI 4.7–34.4) and age ≤1 month (aOR 8.7; 95% CI 3.0–24.6) were the strongest risk factors for HA-MRSA (Table 4). Living in long-term care facilities (LTCFs) (aOR 5.2; 95% CI 1.5–17.4), antibiotic use within two months of the current SAB episode (aOR 5.1; 95% CI 2.8–9.1), hospital stay of ≥13 days before the current SAB episode (aOR 2.8; 95% CI 1.3–5.6) and mechanical ventilation (aOR 2.2; 95% CI 1.07–4.6) were also independent risk factors of HA-MRSA infection. Compared to those aged 65 years and older, patients aged between 25–34 years had 70% reduced odds of HA-MRSA infection in the multivariable model (aOR 0.3; 95% CI 0.08–0.7). The Hosmer-Lemeshow's goodness-of-fit test indicated that our model fit the data well (p = 0.123).

**Table 4:** Multivariable analysis of risk factors for HA-MRSA infection in comparison to HA-MSSA infection among hospitalised patients in Gauteng and Western Cape during 2014.

Characteristic	aOR	95% CI	p value
Burns	12.7	4.7–34.4	<0.001
Age (≤ 1 month vs ≥65 years)	8.7	3.0–24.6	<0.001
Resident in LTCF^a^	5.2	1.5–17.4	0.008
Previous antibiotic use^b^	5.1	2.8–9.1	<0.001
LOS before positive culture (≥13 days vs 0–4 days)	2.8	1.3–5.6	0.004
Mechanical ventilation^c^	2.2	1.07–4.6	0.031
Age (25–34 years vs ≥65 years)	0.3	0.08–0.7	0.016
Sex (Male)^d^	1.3	0.7–2.2	0.443

Hosmer–Lemeshow goodness-of-fit p value = 0.1231. aOR, adjusted odds ratio; CI, confidence interval; LOS, length of hospital stay.

^a^ Within a year of the current culture;

^b^ Two months prior the current culture;

^c^ On the day of positive culture.

^d^ Although sex was not a significant risk factor in the univariate analysis, it was kept in the multivariable model to control for possible confounding.

## Discussion

In our study, we found a high prevalence of MRSA, and HA-MRSA accounted for approximately a third of *S*. *aureus* bloodstream infections among patients admitted to tertiary public-sector hospitals in Gauteng and Western Cape. Patients infected with HA-MRSA had poorer outcomes such as higher mortality and longer hospital stays, compared to patients infected with HA-MSSA. Patients with burns, neonates and who lived at LTCFs were at highest risk of developing HA-MRSA infection, and the duration of hospitalisation, mechanical ventilation, and antibiotic exposure were also significant risk factors, but of lower magnitude.

In our study, the prevalence of MRSA among patients with SAB was lower than previously reported in similar South African studies [6,10]. Although differences in geographic regions where studies were conducted and different patient populations could have resulted in the different MRSA rates, other prevalence and incidence studies conducted in South Africa have found a decline in MRSA [11,12]. Lower MRSA rates are likely due to improved infection prevention strategies, diagnostic stewardship activities, and accurate recording of test results in laboratory information systems. Nonetheless, the rate of MRSA among current hospitals remains high compared to the recently published rate of 24% for South Africa, and indicates that control efforts should be strengthened in order to reduce infections [13].

Similar to previous studies, we found that compared to patients with HA-MSSA infections, a higher proportion of patients with HA-MRSA had longer hospital stays and higher mortality rates [14,15]. These results suggest that MRSA could be pre-disposing patient to poor outcomes, although this is difficult to ascertain due to other co-morbidities and differences in treatment practices. Nonetheless, the adverse role of MRSA in patient outcomes has been demonstrated in published meta-analyses showing strong evidence that MRSA bacteraemia is independently associated with death, despite the presence of co-morbidities [4,16]. Thus, it is likely that reduction of MRSA bacteraemia, especially in hospital settings, will result in less adverse events and better outcomes for patients.

Control of MRSA in healthcare settings relies on understanding factors that predispose patients to the acquisition. Risk factors for HA-MRSA acquisition have been well described and include, invasive procedures, long hospital stay, antibiotic exposure and use of medical devices [17–19]. The strongest risk factor for HA-MRSA infection in our setting was having burns. To the best of our knowledge, this is the first multi-centre study that included patients of all ages admitted in a variety of wards to demonstrate this independent association. The majority of studies describing the impact of HA-MRSA on burn patients have limited the study population to only patients with burns and/or burn units [15,20–22]. Previous studies done in two tertiary hospitals in South African provinces found that *S*. *aureus* was the major pathogen infecting patients in burn units, and MRSA accounted for 66% of these infections in KwaZulu-Natal Province and 58% in Eastern Cape Province [23,24]. A similar study of patients with bloodstream infections and severe burns conducted in Gauteng found that 35% of these infections were due to MRSA [25]. Taken together, these results demonstrate that patients with burns in public hospitals are an important sub-population with increased risk of HA-MRSA.

Our study showed that neonates represented the highest proportion of patients infected with HA-MRSA. In addition, neonates had eight times the odds of HA-MRSA infection compared to HA-MSSA. These findings are contrary to studies done in Scotland, Belgium and the USA that demonstrated a higher incidence of HA-MRSA infections among adults, and an association of HA-MRSA with older age [26–28]. The World Health Organization (WHO) has reported that there is a higher burden of hospital-associated infections in low- and middle-income countries, and as opposed to adults in high-income countries, neonates are most at risk in low- and middle-income countries, with up to 20 times higher infection rates [1]. Thus, our findings support those of WHO and emphasise the need to control MRSA acquisition in neonates as they contribute the most to the burden of disease and are likely to have unfavourable outcomes.

Residents of LTCFs, which are often older frail patients with underlying conditions, were also identified as a subset of hospitalised individuals independently associated with HA-MRSA. Similar to our study, one laboratory-based study including patients of all ages found that MRSA positivity was strongly associated (aOR 3.53; 95% CI 2.79–4.46) with residents of LTCFs compared to patients in acute care hospitals or other facilities such as military hospitals [28]. LTCFs are known as “MRSA reservoirs” due to high colonisation rates of up to 58% in some instances [29]. Colonisation, coupled with the presence of other illnesses, is important as it increases the risk of MRSA infection both at LTCFs and when patients are hospitalised [30]. Thus, our finding supports the conclusion that LTCFs are important sources of MRSA and residents of these facilities are more likely to be infected with HA-MRSA upon hospitalisation.

In our study, previous antibiotic exposure independently increased the odds of HA-MRSA five-fold compared to HA-MSSA. Previous studies have similarly found that antibiotic exposure is a significant risk factor for HA-MRSA infection [18,19,29]. A main strategy to tackle antimicrobial resistance in healthcare settings is through antimicrobial stewardship programmes. However, South African guidelines for stewardship programmes have only recently been published and not fully implemented in public-sector hospitals [31]. Although limited to two wards, an antibiotic stewardship programme successful in reducing antibiotic priscribing was reported by one of the current hospitals [32], suggesting that widespread implemetation of these interventions is likely to reduce antibiotic consumption and infections with antibiotic resistant organisms [33].

We found that the odds of HA-MRSA infection were twice as likely as HA-MSSA when patients were mechanically ventilated, underlining the importance of the presence of medical devices in hospital acquisition of drug-resistant organisms. In the United States, ventilator-associated pneumonia accounts for 39.1% of pneumonia events [34]. In addition, ventilator-associated pneumonia is the leading device-associated infection in China and Turkey [35,36].

Similar to other studies, we also found that HA-MRSA infection is associated with an overall longer hospital stay [6,7,27], and hospital stay of over two weeks increased the odds of HA-MRSA infection three-fold compared to HA-MSSA. These findings demonstrate a reciprocal relationship between MRSA and hospital stay, where hospital stay increases the risk of MRSA infection and MRSA infection lengthens hospital stay. Reasons for infection among patients with lengthy hospital stays include severe illness, use of medical devices and constant and prolonged exposure to healthcare workers and/or other patients that may be colonised or infected with MRSA.

One of the strengths of our study was the use of enhanced surveillance data that were extensive and of good quality. Additionally, we were able to identify important risk factors for HA-MRSA not described in previous South African studies. However, the risk factors described in our study may not reflect risk factors for patients admitted to non-tertiary public-sector hospitals in South Africa. Due to the small number of patients with burns, neonates and LTCFs residents who were found to be at high risk of HA-MRSA, our analysis lacked the statistical power to find any association with known hospital-related factors specific to these patients. Hospital-related factors such as overcrowding, hand hygiene practices, and the existence of antimicrobial stewardship programmes are important determinants of HA-MRSA that were not collected by the surveillance programmes from which we obtained data. Thus, we could not determine their contribution to HA-MRSA acquisition. This information is critical for prevention of HA-MRSA infection and other hospital-related infections.

## Conclusions

The prevalence of MRSA among patients with SAB hospitalised in the five tertiary-level public-sector hospitals in South Africa remains higher than expected. In view of our findings that patients with burns, neonates, those admitted in LTCFs, prolonged hospitalisation, mechanical ventilation, and antibiotic exposure are important risk factors for HA-MRSA infection, control strategies should employ use of “MRSA bundles”. These bundles may include optimum hand hygiene, educating healthcare workers on best practices for handling ventilated patients, isolation and cohorting of infected patients, antimicrobial stewardship programmes, surveillance screening, and decolonisation of high risk patients [37–41]. In order to control HA-MRSA, a holistic approach, which includes a combination of these strategies should be adopted in public-sector hospitals.

## References

[1] World Health Organization. Report on the burden of endemic health care-associated infection worldwide. [Internet]. WHO Library Cataloguing-in-Publication Data Geneva; 2011 [cited 2017 Apr 10]. Available from: http://apps.who.int/iris/bitstream/10665/80135/1/9789241501507_eng.pdf

[2] Luzzaro F, Ortisi G, Larosa M, Drago M, Brigante G, Gesu G. Prevalence and epidemiology of microbial pathogens causing bloodstream infections: results of the OASIS multicenter study. Diagn Microbiol Infect Dis. 2011; 69(4):363–9. doi: 10.1016/j.diagmicrobio.2010.10.016 21396530 10.1016/j.diagmicrobio.2010.10.016

[3] Stefani S, Chung DR, Lindsay JA, Friedrich AW, Kearns AM, Westh H, et al Meticillin-resistant *Staphylococcus aureus* (MRSA): global epidemiology and harmonisation of typing methods. Int J Antimicrob Agents. 2012 4; 39(4):273–82. doi: 10.1016/j.ijantimicag.2011.09.030 22230333 10.1016/j.ijantimicag.2011.09.030

[4] Cosgrove SE, Sakoulas G, Perencevich EN, Schwaber MJ, Karchmer AW, Carmeli Y. Comparison of mortality associated with methicillin-resistant and methicillin-susceptible *Staphylococcus aureus* bacteremia: a meta-analysis. Clin Infect Dis. 2003; 36(1):53–9. doi: 10.1086/345476 12491202 10.1086/345476

[5] Whitby M, McLaws ML, Berry G. Risk of death from methicillin-resistant *Staphylococcus aureus* bacteraemia: a meta-analysis. Med J Aust. 2001; 175(5):264–7. 11587259 10.5694/j.1326-5377.2001.tb143562.x

[6] Fortuin-de Smidt MC, Singh-moodley A, Badat R, Quan V, Kularatne R, Nana T, et al *Staphylococcus aureus* bacteraemia in Gauteng academic hospitals, South Africa. Int J Infect Dis. 2015; 30:41–8. doi: 10.1016/j.ijid.2014.10.011 25448331 10.1016/j.ijid.2014.10.011

[7] Thampi N, Showler A, Burry L, Bai AD, Steinberg M, Ricciuto DR, et al Multicenter study of health care cost of patients admitted to hospital with *Staphylococcus aureus* bacteremia: impact of length of stay and intensity of care. Am J Infect Control. 2015; 43(7):739–44. doi: 10.1016/j.ajic.2015.01.031 25769617 10.1016/j.ajic.2015.01.031

[8] Antonanzas F, Lozano C, Torres C. Economic features of antibiotic resistance: the case of methicillin-resistant *Staphylococcus aureus*. Pharmacoeconomics. 2014; 33(4):285–325. doi: 10.1007/s40273-014-0242-y 25447195 10.1007/s40273-014-0242-y

[9] Wayne P. Clinical and Laboratory Standards Institute. Performance standards for anti-microbial susceptibility testing: twenty-third informational supplement. CLSI document M100-S24 (2014).

[10] Perovic O, Iyaloo S, Kularatne R, Lowman W, Bosman N, Wadula J, et al Prevalence and trends of *Staphylococcus aureus* bacteraemia in hospitalized patients in South Africa, 2010 to 2012: laboratory-based surveillance mapping of antimicrobial resistance and molecular epidemiology. PLoS One. 2015; 10(12):e0145429 doi: 10.1371/journal.pone.0145429 26719975 10.1371/journal.pone.0145429 PMC4697812

[11] Falagas ME, Karageorgopoulos DE, Leptidis J, Korbila IP. MRSA in Africa: filling the global map of antimicrobial resistance. PLoS One. 2013; 8(7):e68024 doi: 10.1371/journal.pone.0068024 23922652 10.1371/journal.pone.0068024 PMC3726677

[12] Naidoo R, Nuttall J, Whitelaw A, Eley B. Epidemiology of *Staphylococcus aureus* bacteraemia at a tertiary children’s hospital in Cape Town, South Africa. PLoS One. 2013; 8(10):e78396 doi: 10.1371/journal.pone.0078396 24167621 10.1371/journal.pone.0078396 PMC3805599

[13] Zigmond J, Pecan L, Hájek P, Raghubir N, Omrani AS. MRSA infection and colonization rates in Africa and Middle East: a systematic review & meta-analysis. Int J Infect Dis. 2014; 21S:391 doi: 10.1016/j.ijid.2014.03.1227

[14] Campbell RS, Emons MF, Mardekian J, Girgenti D, Gaffney M, Yu H. Adverse clinical outcomes and resource utilization associated with methicillin-resistant and methicillin-sensitive *Staphylococcus aureus* infections after elective surgery. Surg Infect (Larchmt). 2015; 16(5):543–52. doi: 10.1089/sur.2013.250 26125541 10.1089/sur.2013.250

[15] Issler-Fisher AC, McKew G, Fisher OM, Harish V, Gottlieb T, Maitz PKM. Risk factors for, and the effect of MRSA colonization on the clinical outcomes of severely burnt patients. Burns. 2015; 41(6):1212–20. doi: 10.1016/j.burns.2015.03.003 26150350 10.1016/j.burns.2015.03.003

[16] Kaasch AJ, Barlow G, Edgeworth JD, Fowler VG, Hellmich M, Hopkins S, et al *Staphylococcus aureus* bloodstream infection: a pooled analysis of five prospective, observational studies. J Infect. 2014; 68(3):242–51. doi: 10.1016/j.jinf.2013.10.015 24247070 10.1016/j.jinf.2013.10.015 PMC4136490

[17] Carnicer-Pont D, Bailey KA, Mason BW, Walker AM, Evans MR, Salmon RL. Risk factors for hospital-acquired methicillin-resistant *Staphylococcus aureus* bacteraemia: a case-control study. Epidemiol Infect. 2006; 134(6):1167–73. doi: 10.1017/S0950268806006327 16623990 10.1017/S0950268806006327 PMC2870517

[18] Graffunder EM, Venezia RA. Risk factors associated with nosocomial methicillin-resistant *Staphylococcus aureus* (MRSA) infection including previous use of antimicrobials. J Antimicrob Chemother. 2002; 49(6):999–1005. 12039892 10.1093/jac/dkf009

[19] Oztoprak N, Cevik MA, Akinci E, Korkmaz M, Erbay A, Eren SS, et al Risk factors for ICU-acquired methicillin-resistant *Staphylococcus aureus* infections. Am J Infect Control. 2006; 34(1):1–5. doi: 10.1016/j.ajic.2005.07.005 16443085 10.1016/j.ajic.2005.07.005

[20] Safdar N, Marx J, Meyer NA, Maki DG. Effectiveness of preemptive barrier precautions in controlling nosocomial colonization and infection by methicillin-resistant *Staphylococcus aureus* in a burn unit. Am J Infect Control. 2006; 34(8):476–83. doi: 10.1016/j.ajic.2006.01.011 17015152 10.1016/j.ajic.2006.01.011

[21] Kaiser ML, Thompson DJ, Malinoski D, Lane C, Cinat ME. Epidemiology and risk factors for hospital-acquired methicillin-resistant *Staphylococcus aureus* among burn patients. J Burn Care Res. 2011; 8(1):136–9. doi: 10.1097/BCR.0b013e318217f92d PMID: 21422940 10.1097/BCR.0b013e318217f92d 21422940

[22] Abbasi-Montazeri E, Khosravi AD, Feizabadi MM, Goodarzi H, Khoramrooz SS, Mirzaii M, et al The prevalence of methicillin resistant *Staphylococcus aureus* (MRSA) isolates with high-level mupirocin resistance from patients and personnel in a burn center. Burns. 2013; 39(4):650–4. doi: 10.1016/j.burns.2013.02.005 23499497 10.1016/j.burns.2013.02.005

[23] Bhat VG, Vasaikar SD, Bauer K. Bacteriological profile and antibiogram of aerobic burn wound isolates in Mthatha, Eastern Cape, South Africa. South Afr J Epidemiol Infect. 2010; 25(4):16–9. doi: 10.1080/10158782.2010.11441404

[24] Swe Swe-Han K, Coovadia Y. Prevalence of antimicrobial resistant bacteria from adult ICUs and the Burns unit at a large tertiary hospital in Durban. Int J Infect Control. 2010; 6(2):2–9. doi: 10.3396/ijic.V6i2.015.10

[25] Bahemia IA, Muganza A, Moore R, Sahid F, Menezes CN. Microbiology and antibiotic resistance in severe burns patients: a 5 year review in an adult burns unit. Burns. 2015; 41(7):1536–42. doi: 10.1016/j.burns.2015.05.007 26051799 10.1016/j.burns.2015.05.007

[26] Kallen AJ, Mu Y, Bulens S, Reingold A, Petit S, Gershman K, et al Health care-associated invasive MRSA infections, 2005–2008. JAMA. 2010; 304(6):641–8. doi: 10.1001/jama.2010.1115 20699455 10.1001/jama.2010.1115

[27] Lawes T, Edwards B, Lopez-Lozano J-M, Gould I. Trends in *Staphylococcus aureus* bacteraemia and impacts of infection control practices including universal MRSA admission screening in a hospital in Scotland, 2006–2010: retrospective cohort study and time-series intervention analysis. BMJ Open. 2012; 2(3):e000797 doi: 10.1136/bmjopen-2011-000797 22685226 10.1136/bmjopen-2011-000797 PMC3378947

[28] Catry B, Latour K, Jans B, Vandendriessche S, Preal R, Mertens K, et al Risk factors for methicillin resistant *Staphylococcus aureus*: a multi-laboratory study. PLoS One. 2014; 9(2):e89579 doi: 10.1371/journal.pone.0089579 24586887 10.1371/journal.pone.0089579 PMC3935888

[29] Stone ND, Lewis DR, Johnson TM, Hartney T, Chandler D, Byrd-Sellers J, et al Methicillin-resistant *Staphylococcus aureus* (MRSA) nasal carriage in residents of Veterans Affairs long-term care facilities: role of antimicrobial exposure and MRSA acquisition. Infect Control Hosp Epidemiol. 2012; 33(6):551–7. doi: 10.1086/665711 22561709 10.1086/665711

[30] Cheng VC, Tai JW, Wong ZS, Chen JH, Pan KB, Hai Y, et al Transmission of methicillin-resistant *Staphylococcus aureus* in the long term care facilities in Hong Kong. BMC Infect Dis. 2013; 13(1):205 doi: 10.1186/1471-2334-13-205 23641974 10.1186/1471-2334-13-205 PMC3651730

[31] Mendelson M, Matsoso MP. The South African antimicrobial resistance strategy framework [Internet]. Monitoring, surveillance and National Plans. 2015 [cited 2017 Jan 11].54–61. Available from: http://www.fidssa.co.za/Content/Documents/2015_01.pdf

[32] Boyles TH, Whitelaw A, Bamford C, Moodley M, Bonorchis K, Morris V, et al Antibiotic stewardship ward rounds and a dedicated prescription chart reduce antibiotic consumption and pharmacy costs without affecting inpatient mortality or re-admission rates. PLoS One. 2013; 8(12):1–7. doi: 10.1371/journal.pone.0079747 24348995 10.1371/journal.pone.0079747 PMC3857167

[33] Brink AJ, Messina AP, Feldman C, Richards GA, Becker PJ, Goff DA, et al Antimicrobial stewardship across 47 South African hospitals: an implementation study. Lancet Infect Dis. 2016;pii: S1473–3099(16)30012–3. doi: 10.1016/S1473-3099(16)30012-3 27312577 10.1016/S1473-3099(16)30012-3

[34] Magill SS, Edwards JR, Bamberg W, Beldavs ZG, Dumyati G, Kainer MA, et al Multistate point-prevalence survey of health care–associated infections. N Engl J Med. 2014; 370(13):1198–208. doi: 10.1056/NEJMoa1306801 24670166 10.1056/NEJMoa1306801 PMC4648343

[35] Hu B, Tao L, Rosenthal VD, Liu K, Yun Y, Suo Y, et al Device-associated infection rates, device use, length of stay, and mortality in intensive care units of 4 Chinese hospitals: International Nosocomial Control Consortium findings. Am J Infect Control. 2013; 41(4):301–6. doi: 10.1016/j.ajic.2012.03.037 23040491 10.1016/j.ajic.2012.03.037

[36] Tekin R, Dal T, Pirinccioglu H, Oygucu SE. A 4-year surveillance of device-associated nosocomial infections in a neonatal intensive care unit. Pediatr Neonatol. 2013; 54(5):303–8. doi: 10.1016/j.pedneo.2013.03.011 23643153 10.1016/j.pedneo.2013.03.011

[37] Nelson MU, Gallagher PG. Methicillin-resistant *Staphylococcus aureus* in the neonatal intensive care unit. Semin Perinatol. 2012; 36(6):424–30. doi: 10.1053/j.semperi.2012.06.004 23177801 10.1053/j.semperi.2012.06.004 PMC3508470

[38] Popoola VO, Budd A, Wittig SM, Ross T, Aucott SW, Perl TM, et al Methicillin-resistant *Staphylococcus aureus* transmission and infections in a neonatal intensive care unit despite active surveillance cultures and decolonization: challenges for infection prevention. Infect Control Hosp Epidemiol. 2014; 35(4):412–8. doi: 10.1086/675594 24602947 10.1086/675594 PMC3950943

[39] Barbut F, Yezli S, Mimoun M, Pham J, Chaouat M, Otter JA. Reducing the spread of *Acinetobacter baumannii* and methicillin-resistant *Staphylococcus aureus* on a burns unit through the intervention of an infection control bundle. Burns. 2013; 39(3):395–403. doi: 10.1016/j.burns.2012.07.007 22884127 10.1016/j.burns.2012.07.007

[40] Morrill HJ, Caffrey AR, Jump RLP, Dosa D, LaPlante KL. Antimicrobial stewardship in long-term care facilities: a call to action. J Am Med Dir Assoc. 2016; 17(2):183.e1–183.e16. doi: 10.1016/j.jamda.2015.11.013 26778488 10.1016/j.jamda.2015.11.013 PMC6348872

[41] Traa MX, Barboza L, Doron S, Snydman DR, Noubary F, Nasraway SA. Horizontal infection control strategy decreases methicillin-resistant *Staphylococcus aureus* infection and eliminates bacteremia in a surgical ICU without active surveillance. Crit Care Med. 2014; 42(10):2151–7. doi: 10.1097/CCM.0000000000000501 24979485 10.1097/CCM.0000000000000501

